# Designing a test battery for workers’ well-being: the first wave of the Tsukuba Salutogenic Occupational Cohort Study

**DOI:** 10.1265/ehpm.23-00372

**Published:** 2024-08-02

**Authors:** Shotaro Doki, Daisuke Hori, Tsukasa Takahashi, Kei Muroi, Mami Ishitsuka, Asako Matsuura, Hotaka Tsukada, Wakako Migaki, Norishige Kanai, Yu Ikeda, Soshi Takao, Ichiyo Matsuzaki, Shin-ichiro Sasahara

**Affiliations:** 1 Institute of Medicine, University of Tsukuba, 1-1-1 Tennodai, Tsukuba, Ibaraki 305-8575, Japan; 2 Occupational Health Committee, Tsukuba Science City Network, 2-20-5 Takezono, Tsukuba, Ibaraki 305-0032, Japan; 3 Graduate School of Comprehensive Human Sciences, University of Tsukuba, 1-1-1 Tennodai, Tsukuba, Ibaraki 305-8575, Japan; 4 School of Nursing at Narita, International University of Health and Welfare, 4-3 Kozinomori, Narita, Chiba 286-8686, Japan; 5 Division of Translational Nursing, Toho University, 2-2-1 Miyama, Funabashi, Chiba 274-8510, Japan; 6 Department of Epidemiology, Graduate School of Medicine, Dentistry and Pharmaceutical Sciences, Okayama University, Okayama, Okayama 700-8558, Japan; 7 International Institute for Integrative Sleep Medicine, University of Tsukuba, 1-1-1 Tennodai, Tsukuba, Ibaraki 305-8575, Japan

**Keywords:** Mental health, Psychometric, Stress, Test battery, Well-being, Worker

## Abstract

**Background:**

In recent years, there has been a diversification of working styles. The concept of workers’ well-being is no longer limited to material wealth, such as how satisfied they are with their wages. Psychological enrichment, encompassing factors like motivation and interpersonal relationships, is also important. This study was established to develop a scale to evaluate the well-being of workers, named the Abundance Index for Workers (AIW). This new concept proposed by the authors comprehensively considers both job-related resources and personal and societal resources.

**Methods:**

This study was carried out as part of the Tsukuba Salutogenic Occupational Cohort Study (T-SOCS). We utilized data from a survey targeting workers affiliated with institutions under the Tsukuba Science City Network, examining aspects of their daily life, work, and mental health. The deviation scores for each survey item were averaged to calculate an overall score. The correlations of the comprehensive score with the Patient Health Questionnaire-9 (PHQ-9) index for depression and the Single-item Presenteeism Question (SPQ) index for presenteeism were analyzed to determine criterion-related validity.

**Results:**

The number of participants analyzed was 2,745. Factor analysis categorized the data into three factors: workplace mental health, societal resources, and lifestyle habits. Cronbach’s α coefficient was 0.688. There were correlations of −0.363 (p < 0.001) between the total score and SPQ, and −0.558 (p < 0.001) between the total score and PHQ-9, suggesting a degree of criterion-related validity.

**Conclusions:**

In this study, we designed a test battery that assesses workers’ well-being based on job-related resources and the richness of societal resources. The internal consistency of this battery was not as high as expected due to the broad scope of well-being. Although it is difficult to evaluate workers’ well-being on a single scale, we believe that the AIW functions well as a test battery by combining scales with different attributes, which enables well-being to be captured from as many different perspectives as possible. This tool is designed to assist individuals in evaluating their own well-being and recognizing factors that can enhance it.

**Trial registration:**

Not applicable; this study is not an intervention trial.

## Background

In recent years, there has been a diversification of working styles, leading to a shift in the conceptualization of worker well-being. This conceptualization extends beyond mere financial or material satisfaction, encompassing psychological aspects such as motivation and interpersonal relationships. The World Health Organization (WHO) foregrounds this psychological perspective by defining health as “a state of complete physical, mental and social well-being and not merely the absence of disease or infirmity,” further highlighting the integral role of mental health [1].

It is essential to obtain a comprehensive understanding of workers’ well-being in order to support workers’ mental health. The concept of workers’ well-being takes into account resources related to work and life, as well as individual and societal resources. This well-being pertains to a healthy psychological state, and feelings of happiness, satisfaction, and internal stability. Because private life is related to job satisfaction and work-related happiness is related to happiness in one’s private life [2], it is not possible to understand a worker’s well-being by considering work alone or private life alone, so an approach involving both private life and work is needed. The National Institute for Occupational Safety (NIOSH) also evaluates workers’ well-being comprehensively, considering not only the work environment but also connections with society, health status, home, community, and broader societal metrics [3].

Since a test battery is appropriate for assessing attributes with multidimensional diversity [4], we used a test battery to measure workers’ well-being. When considering an individual’s sense of well-being, besides the workplace environment, factors like residential environment, connections with society, health status, and the joy and interest in life play pivotal roles, as these have been significantly associated with life satisfaction [5]. It has also been indicated that there are positive relationships between job satisfaction and life satisfaction, happiness, positive emotions, and the absence of negative emotions [6]. Human well-being comprises a broader array of states and outcomes, encompassing not just mental and physical health, but also happiness, life satisfaction, meaning and purpose, traits and virtues, and intimate social relationships [7].

While numerous scales are available to assess well-being and mental health, to the best of our knowledge, there is no specific measure designed to evaluate the well-being of workers using a test battery. Because workers’ well-being is influenced by factors such as individual lifestyle, environment, social relationships, career, and job roles, it is challenging to comprehensively assess a worker’s well-being using a single indicator. In the fields of psychiatry and psychology, it is common to employ a test battery to comprehensively assess personality traits. A test battery is used to evaluate various aspects and abilities related to a specific purpose or theme. It consists of a combination of multiple different assessment tools and tests. For example, cognitive test batteries have been used in the evaluation of patients with depression [8, 9].

This study was established to develop a unique test battery called the Abundance Index for Workers (AIW) based on existing evaluation methods, taking into consideration workers’ characteristics, sources of stress in daily life, and diverse working styles. This new tool for assessing workers’ well-being integrates not just job-related resources, but also personal and societal resources.

## Methods

This study was conducted as part of the Tsukuba Salutogenic Occupational Cohort Study (T-SOCS). The T-SOCS is a survey concerning lifestyle, work, and mental health targeting 21,875 workers employed by institutions affiliated with the Tsukuba Science City Network in and around Tsukuba City, Ibaraki Prefecture, Japan. The T-SOCS study is a survey targeting all workers at institutions affiliated with the Tsukuba Science City Network. Workers were invited to participate via email. The present study is cross-sectional in nature because we used data from the first wave of T-SOCS performed in 2022.

### Test battery development

The T-SOCS questionnaire was developed by the Occupational Health Committee Working Group, composed of occupational health experts. The content of the questions was formulated with reference to prior research conducted by the Tsukuba Science City Network [10–15]. In T-SOCS, we extensively investigated factors such as the living environment in one’s area of residence, lifestyle-related factors like smoking, alcohol consumption, and exercise, individual stress-coping abilities, depression, feelings of well-being, sleep, social capital in communities and workplaces, and occupational stress.

Among these, we specifically selected items related to work, personal, and societal resources to formulate the questions for our test battery. The test battery items include smoking [16], alcohol consumption [16], disease status [16], exercise habits, working hours, commuting time, harassment in the workplace [17], positivity, job satisfaction [18], gratitude scale at work [19], workplace social capital [20], the Utrecht Work Engagement Scale [21, 22], community social capital (trust in and attachment to one’s community) [23], and stress-coping ability (sense of coherence: SOC) [24]. Since the distributions of each item vary, we standardized each individual item and subsequently calculated the mean.

The deviation scores for each item, calculated as

[(X−Xaverage)×10/Standard deviation]+50,

were averaged to determine the overall score.

### Validity and reliability assessment

#### Participants

We invited 21,875 workers employed by the institutions affiliated with the Tsukuba Science City Network to participate in this study via email. The number of respondents was 3,860 (17.6%), 2,745 (12.5%) of whom agreed to cooperate in the survey. 346 respondents declined to participate in this study. The number of missing data entries was 769. The valid response rate was 71.1%. Table 1 shows the characteristics of the participants in this study. The mean age was 46.3 years (standard deviation: 11.6). The predominant occupational category was researcher, containing 1,115 individuals (40.6%), which is a unique feature of this study.

**Table 1:** Basic socio-demographic characteristics of participants (N = 2,745)

		**N**	**%**	**Mean (SD)**
Age (year)*		2,730	99.5	46.3 (11.6)
Sex	Male	1,616	58.9	
	Female	1,101	40.1	
	Others	4	0.1	
	No response	24	0.9	
Marital status	Single	620	22.6	
	Married/partner	2,019	73.6	
	Widowed/divorced	102	3.7	
	No response	4	0.1	
Type of job	Administrative	910	33.2	
	Research	1,115	40.6	
	Technical	294	10.7	
	Professional	238	8.7	
	Others	188	6.8	

SD: Standard deviation; *: Fifteen participants were reluctant to disclose their age.

#### Validity

For construct validity, the researchers discussed determining which items should be employed in the index. Items with high inter-item correlations (those with Pearson’s correlation coefficient exceeding 0.5) and items with factor loadings lower than 0.4 in the factor analysis (using the Varimax rotation method) were excluded. Factors with eigenvalue greater than 1 were used to determine the number of components according to the Kaiser criterion. We created a path diagram between factors using Structural Equation Modeling (SEM). We examined criterion-related validity by determining the correlation between the total score and an indicator of the severity of depression, the Patient Health Questionnaire-9 (PHQ-9) [25], and an indicator of presenteeism, the Single-item Presenteeism Question (SPQ) [26]. The SPQ has a scoring range of 0–99, with a higher score indicating higher presenteeism.

#### Reliability

To assess reliability, we calculated Cronbach’s α coefficient. We considered a Cronbach’s α coefficient of 0.6 or higher to indicate satisfactory reliability [27].

### Statistical analysis

For statistical analyses, we utilized IBM SPSS 29 and IBM SPSS AMOS (IBM Corp., Armonk, NY).

### Ethical approval

This study received approval from the Ethical Committee of the Faculty of Medicine at Tsukuba University (No. 1669).

## Results

**Fig. 1:**
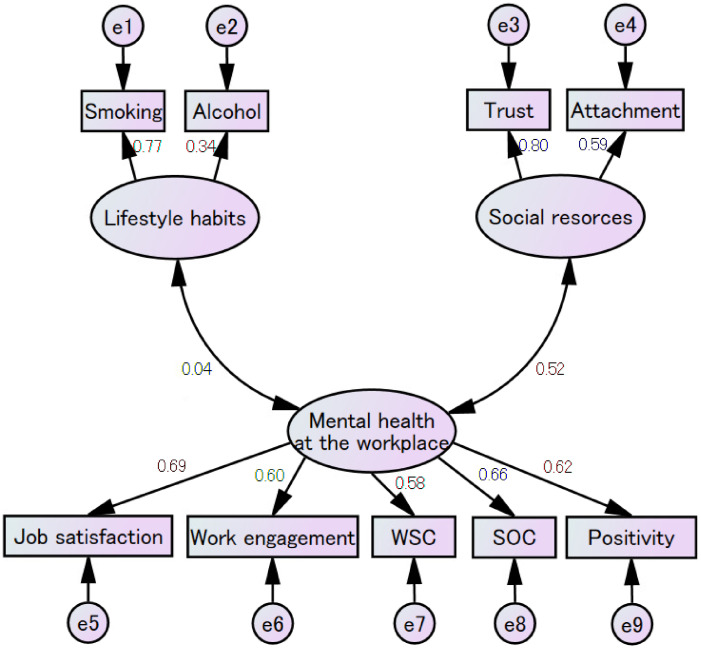
Path diagram between factors, as determined by Structural Equation Modeling. Rectangles are observed variables; ellipses are latent variables; e1 to e9 are error variables; chi-square 259.4 (d.f. 25), p < 0.001; comparative fit index, 0.951; root mean square error of approximation, 0.049 (90% confidence interval 0.044–0.055).

**Fig. 2:**
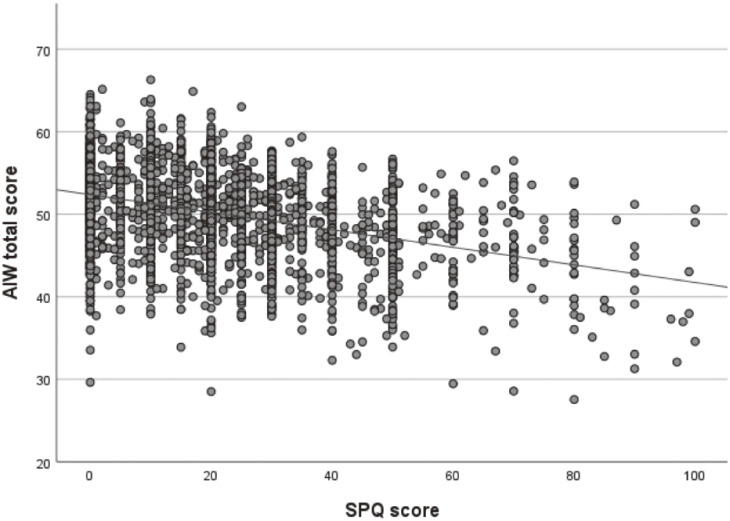
Scatter plot and correlation between AIW total score and SPQ score. Pearson’s correlation coefficient between the total score of AIW and SPQ was −0.363 (p < 0.001).

**Fig. 3:**
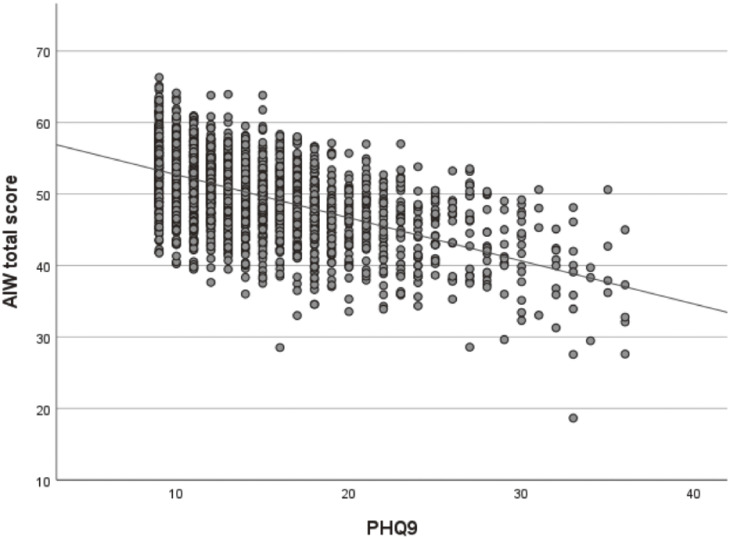
Scatter plot and correlation between AIW total score and HPQ-9. Pearson’s correlation coefficient between the total score of AIW and HPQ-9 was −0.558 (p < 0.001).

The range of the absolute values of Pearson’s correlation coefficients for each item was from ≤0.01 to 0.49. A significant correlation was observed between the gratitude scale at work and workplace social capital (Pearson’s correlation coefficient 0.618, p < 0.001). Taking into account the similarity between the question items, we excluded the gratitude scale at work from the questionnaire items. As a result of the factor analysis, items such as trustworthiness, illness, exercise, commuting, working hours, and harassment, which had factor loadings of less than 0.4, were excluded. Upon conducting the factor analysis again, the questionnaire items were classified into three categories: mental health at the workplace, social resources, and lifestyle habits (Table 2). The factor analysis and the rotation of the component matrix indicated that three factors explained 39% of the variance, as shown in Table 3. Cronbach’s alpha coefficient was 0.688. The path diagram between factors, as determined by SEM, is shown in Fig. 1 (comparative fit index = 0.951; root mean square error of approximation = 0.049). Scatter plots of the total scores of the test battery plotted against SPQ and PHQ-9 are shown in Figs. 2 and 3, respectively. Pearson’s correlation coefficient between the total score and SPQ was −0.363 (p < 0.001), and that between the total score and PHQ-9 was −0.558 (p < 0.001). Although these correlations are weak to moderate, they do indicate a certain degree of criterion-related validity.

**Table 2:** Factor loadings of the scale

	**Factors**		

	**1**	**2**	**3**
Job satisfaction	**0.715**	0.098	0.030
Sense of coherence	**0.643**	0.174	−0.122
Work engagement	**0.583**	0.137	0.062
Positivity	**0.583**	0.190	0.046
Workplace social capital	**0.542**	0.183	0.031
Trust in one’s community	0.246	**0.748**	−0.019
Attachment to one’s community	0.177	**0.550**	−0.001
Smoking	0.022	0.022	**0.541**
Alcohol consumption	0.005	−0.028	**0.477**

Factor extraction method: Principal axis factoring; Rotation method: Varimax rotation with Kaiser normalization; Kaiser-Meyer-Olkin measure for sampling accuracy was 0.786; Bartlett test of sphericity: p < 0.001 approximate chi-square 4566.739.

**Table 3:** Factor analysis eigenvalues, percentage of variance and cumulative percentage of variance explained

	**Initial eigenvalues**			**Sum of squared loadings after rotation**		

**Component**	**Eigenvalues**	**Percentage of variance (%)**	**Cumulative percentage of variance (%)**	**Eigenvalues**	**Percentage of variance (%)**	**Cumulative percentage of variance (%)**
1	2.958	32.869	32.869	1.991	22.117	22.117
2	1.278	14.197	47.067	0.991	11.010	33.128
3	1.108	12.311	59.378	0.543	6.029	39.156
4	0.771	8.566	67.943			
5	0.736	8.175	76.119			
6	0.647	7.189	83.307			
7	0.552	6.133	89.441			
8	0.499	5.546	94.986			
9	0.451	5.014	100.000			

## Discussion

In this study, we focused on the complex and multidimensional concept of workers’ well-being. We explored how to conceptualize and systematize it in terms of numerous work-related and societal resources, and undertook the development of a new evaluation scale. The notion of workers’ well-being covers a wide range of factors and backgrounds. To capture all of its facets as accurately as possible, we selected and employed question items that reflect its maximum diversity. As a result, and as anticipated, the internal consistency was not high due to the collection of responses to questions on various attributes. This was due to the inherent diversity of the concept of well-being itself.

Evaluating well-being using just one measure is challenging. However, this test battery is designed to capture well-being from multiple perspectives by integrating indices reflecting various attributes. In the UK, the Warwick-Edinburgh Mental Wellbeing Scales have been developed and widely used to measure psychological well-being [28]. To examine well-being more broadly, it is necessary to consider factors related to one’s connection to society and relationships with the local community [29]. Therefore, we have utilized various established assessment tools and created indicators to measure new aspects of well-being in the form of a test battery. Furthermore, we are planning to offer the participants of this study access to the AIW web application. We hope that, similar to an assessment of stress levels among employees that is legally mandated to the employers in Japan, by enabling accurate self-assessment of the different components of one’s own well-being, this battery will contribute to enhancing well-being.

We observed significant weak to moderate correlations between the total score of AIW and measures of depression and presenteeism, suggesting a certain validity and potential utility of the scale that we developed. The concept of workers’ well-being is influenced not just by the sheer quantity of resources available to the worker, but also by a multitude of external factors, including cultural background and life satisfaction, which shift over time. Especially in modern society, increasing attention is being placed on how people from different generations and cultural backgrounds prioritize material and psychological well-being [30]. Taking these complex factors into account, further research is essential to refine the definition of well-being in line with historical shifts [31].

Limitations of this study include the inability to conduct a qualitative assessment of workers’ well-being and not accounting for individual differences in the value placed on one’s own work. The notion of well-being might also change with the passage of time. As such, the AIW developed in this study may not necessarily be applicable across different times and cultural backgrounds. Additionally, since 40.6% of the participants are researchers, it is difficult to generalize the findings to people of all professions. In this study, the test-retest method was not conducted for the assessment of reliability; therefore, it needs to be implemented in future research.

## Conclusion

Through this research, a new well-being index for workers, AIW, was developed and its preliminary validity was confirmed. This scale offers a novel approach to understanding the well-being of workers by assessing the abundance of work-related and societal resources that they have at their disposal, and it has the potential to serve as a basis for future workplace evaluations and interventions. However, because the concept of well-being may change over time and vary across different cultures, regular review and adjustment of its definition and scoring criteria are necessary. It is anticipated that future research will be performed to further validate and implement the practical applications of the AIW.

## Abbreviations

AIW: Abundance Index for Workers
NIOSH: National Institute for Occupational Safety & Health
PHQ-9: Patient Health Questionnaire-9
SEM: Structural Equation Modeling
SPQ: Single-item Presenteeism Question
T-SOCS: Tsukuba Salutogenic Occupational Cohort Study
WHO: World Health Organization

## Declarations

### Availability of data and material

Data cannot be shared for privacy or ethical reasons.
